# Miscellaneous

**Published:** 1856-12

**Authors:** 


### Miscellaneous.

Mr. Editor:—I wish you to answer me a few questions.

If a person having only the lower incisors, cuspids and bicuspids; and the second upper molars, wishes you to insert teeth *above*: please tell me the *best* plan your long experience has taught you to do such jobs.

Could such a job be made so as to keep its place when in use, and be useful to the person ?

Do you have such jobs? If so please tell me your plan of operation, or how you do them. Would you do a suction job, or clasp to the two molars ? Please answer and give me such information in regard to it as you think will be useful. K.

ANSWER.

In answering which kind of job would be preferred will depend much on circumstances.

If the patient is an adult, the two molars referred to both sound and healthy, with a healthy mouth in all other respects, I would recommend a job with clasps, for this reason; The job would be more useful in mastication, more easily retained in the mouth and, if properly adjusted, would last many years and be serviceable. In that case I would use a wide plate and wide clasps, passing as nearly around the natural teeth as possible, without having to separate with the file if the dens sapientia should be still remaining.

If the patient should be young with the remaining teeth predisposed to decay, I would by all means recommend a job without clasps, depending on the atmospheric pressure alone.

In all respects excepting mastication a well adjusted suction plate would be preferred.

If the clasp job is used, much of its permanency would depend on the care taken of the mouth, and especially the clasp teeth. The plate should be removed at least three times per day and it and the mouth be thoroughly cleansed with tooth powder, soap and such other substances generally used for the purpose.

When queries are propounded, a full statement of the case should be made namely the age of the patient, general condition of the mouth and remaining teeth, then having a full and correct diagnosis, a definite course of treatment and kind of operation could be laid down,

S.

*Infirmary report of the Ohio College of Dental Surgery from March to October*, 1856. *By* E. Osmond D. D. S. *Resident Dentist.*

| | |
|---|---|
| Operations.—Number of teeth filled with gold | 111 |
| " " " gutta percha | 8 |
| " " " over exposed nerve | 6 |
| " " " to end of fangs | 2 |
| Cases of irregularity | 8 |
| " Salivary Calculus | 6 |
| No. of pivot teeth inserted | 4 |
| " teeth extracted | 504 |
| Amalgam fillings removed | 10 |
| Diseased antrum treated | 1 |
| Full sets of teeth inserted | 3 |
| " upper sets " | 4 |
| Partial upper " " | 6 |
| " lower " " | 1 |
| Operations repaired | 7 |

Remarks.—Seven of the amalgam fillings were removed on account of their influence on adjacent gold fillings, the others on account of the patients suffering from mercurial symptoms.

The patient treated for diseased antrum was a lady of nervous sanguine temperament, inclined to the strumous diathesis. On March 22nd I extracted seven roots of teeth for her. One of them, the left superior canine, had been treated with quick lime, two and a half years ago. For the past two years the left eye has been affected, and she has had great uneasiness in the cheek of the same side. When the tooth was extracted a discharge followed its removal. For sometime afterward, the eye became much worse, but ultimately improved, leaving, however, a constant discharge from the puncta lachrymalia. On the 24th of June she again presented herself at the infirmary, at which time a slight, pale discharge was issuing from the socket of the removed fang. With a probe, I discovered a very small opening pointing posteriorly towards the antrum, From the sensation communicated by the probe, I diagnosed a diseased condition of the antrum, and a carious condition of that portion of the maxilla from which the tooth had been extracted. As the bicuspids and molars were absent, I determined to perforate the antrum through the canine fossa. I accordingly dissected the gum from the buccal and palatal surfaces of the bone and removed the carious portion with the forceps, and then, with a drill directed posteriorly, penetrated the antrum. About a teaspoonful of pale fluid escaped, I injected a solution of chlorinated lime, from time to time into the cavity. The cavity was kept open with a tent till July 16th, when I injected a solution of nitrate of silver, two grains to the ounce, and then allowed it to close. Her eye is much improved, and she has now no uneasiness whatever. The gum has healed perfectly; and she wears on a suction plate, an upper set of teeth minus the central incisors and a lateral.

A number of diseased conditions, not mentioned in the list of operations, were prescribed for, most of which were caused by neglect, or by improper and imperfect operations.

*Report of the Executive Committee of the Mississippi Valley Association of Dental Surgeons.*

TheExecutive committee would respectfully submit the following subjects for discussion at the next meeting of the Association.

The most of them are not new points; but they do refer to modes of practice of comparatively recent introduction; and it is important to have the experience of the profession in regard to them as fully as possible.

It is, probably, more important just at the present time to thoroughly test the modes of practice, now in use, than to introduce much that would be altogether new.

1. Is annealed foil, as now used, preferable to blocks for filling; and does it weld as perfectly as "Crystal Gold"?

2. Is the exalted sensibility of decayed teeth the result of inflammation?

3. What constitutional changes will most predispose the teeth to decay?

4. What has been the course pursued and the success in the treatment of alveolar abscess?

5. How does Crystal Gold stand the test of time?

6. What success has been obtained in fang filling?

7. What is the best manner of filling the fangs of teeth, the nerves of which are dead?

7. Is it desirable as a general rule to preserve the fangs of teeth for the reception of artificial substitutes?

9. What conditions of the mouth should determine us in the choice of material for taking impressions for full upper dentures?

10. What are the indications requiring the extraction of the deciduous teeth?

J. TAFT.<br>J. P. ULREY.<br>J. RICHARDSON. } Committee.

---

Tooth Within a Tooth.—Messrs. Editors:—The following singular case was related to me a few days since by Dr. Geo. Fries of this city as having occured in his practice a few years ago. I am permitted to give it publicity with the sanction of his name, an authority that excludes any doubt as to accuracy of observation or good faith in the statement of facts.

The Doctor was summoned in great haste to see a patient residing some two or three miles in the country. On arriving he found a young man of some eighteen years of age suffering with a most violent toothache located in one of the inferior molars,—which one of them he does not distinctly remember. There was a large crown-cavity in the tooth which seemed on examination to be occupied chiefly by a hard movable substance and which after repeated efforts he removed with the blade of a penknife. It proved to be a *miniature tooth*, perfect in all its proportions. The crown corresponding in its general contour with that of the *parent* tooth and was about the size of a small pea. The roots which were three in number were well developed, distinct, symmetrical, divergent and sharply pointe d

I s general appearance was that of bleached or denuded bone, looking as though it had been macerated for a time. The tooth from which it was taken presented no unusual appearances either before or after the extraction. The anomalous specimen was preserved at the time as a great curiosity but was subsequently, by some chance, lost.

Now, will some adventurous and astute expounder of the laws of development interpret this freak of erratic nature. As for ourselves we adopt the confession of the man, who, among other things, was proverbial for his profane instincts. Meeting on a certain occasion, with a somewhat disastrous casuality, after deliberately contemplating the magnitude of his misfortune and concluding that the provocation was quite beyond his peculiar powers, turned with the air of an injured man to the throng of curious and expectant neighbors, who had collected to witness an unprecedented scene and remarked "It's no use gentlemen I can't do justice to the subject."

Yours &c

Cincinnati Nov. 10 1856 J. RICHARDSON.

---

*Aluminium.*—This metal has been suggested to dentists as a substitute for gold in plate-work. It possesses many properties which would render it very desirable to employ it. In the first place, its specific gravity (2.25) is much below that of the ordinary metals. It is nearly that of glass, and this lightness must, of course, add greatly to the comfort with which plates made of the new metal could be worn. Again, it has no tendency to rust, and is not acted on by any of the ordinary solvents. Sulphur does not affect it, nor will any of the oxy-acids dissolve it, its only solvent being hydrochloric acid. This indifference to chemical agents has been supposed to be due to the formation of an oxyd, a thin film of transparent sapphire, at the instant it comes in contact with the atmosphere. Its fusing point is lower than that of silver, and higher than the melting point of zinc. It is malleable, ductile, and sonorous.

Its present price in England is £3, or fifteen dollars per ounce. This is about 25 per cent. cheaper than gold. In reality, however, the ratio between its price and that of the precious metal now used for plate-work, is much less than this statement would imply. Being so very far below gold, in specific gravity, a given weight of aluminium would go more than eight times as far as the same quantity of gold.

With most of the metals it forms characteristic alloys. With copper it forms a fine gold-colored alloy, which does not appear to tarnish readily. Mixed with gold, a very small proportion of it changes the yellow color of that metal to gray.

For the benefit of those who may wish to experiment with this new metal, we give the formula for a *solder of aluminium.* It is composed of *two* parts of aluminium and *one* of silver—without flux.—*American Journal of Dental Science.*

---

*Poisoning from Swallowing Chloroform.*—The *Philadelphia Medical Examiner* contains an interesting case of death following the ingestion of about one ounce and a half of chloroform, diluted with about the same quantity of water. The patient was an intemperate woman, who swallowed the liquid by mistake, supposing it to be sweet spirit of nitre. The first symptoms were those of intoxication, followed by insensibility, stertorous breathing, slow and feeble pulse, and great contraction of the pupils. She lived for about thirty-six hours, and died asphyxiated, having recovered her senses for several hours before death. The stomach was paler thau usual, except in streaks a quarter of an inch in width, from which it was inferred that the organ had been thrown into folds by the irritation of the chloroform, so that only a portion of its surface had been acted on. The mucous membrane was much softened.

---

*Albinos.*—Having seen it stated in the newspapers some time ago, that a negress in Alabama had given birth to three children, two of whom were white and the other black, we addressed a note to Dr. John H. Hundley, of Mooresville, the attending physician, requesting a statement of the facts. We have been favored with a reply from Dr. H., who informs us that it has been ascertained by microscopic examination of the hair of these two children, that the two white ones were albinos, their hair presenting the negro peculiarity as strongly as that of the black child. Our informant adds, that both parents are full-blooded negroes—that the mother has had fourteen children, five albinos and nine blacks, and that the albinos are as white as any of the Caucasian race.

The conjectures about double paternity and superfœtation are, therefore, in this case at least, set at rest. The advantages of the microscopic test are also here clearly set forth.—*Southern Med. and Surg. Jour.*

---

*Solubility of Gallic Acid in Glycerine*—Mr. Thomas Weaver, Pharmaceutist, of Philadelphia, states in a communication to the Medical Reporter, that according to some experiments performed by him, he finds that gallic acid is soluble in glycerine to the extent of *forty* grains to the ounce. This is contrary to what has recently been stated on the subject.

